# LOX-1-Expressing Immature Neutrophils Identify Critically-Ill COVID-19 Patients at Risk of Thrombotic Complications

**DOI:** 10.3389/fimmu.2021.752612

**Published:** 2021-09-20

**Authors:** Behazine Combadière, Lucille Adam, Noëlline Guillou, Paul Quentric, Pierre Rosenbaum, Karim Dorgham, Olivia Bonduelle, Christophe Parizot, Delphine Sauce, Julien Mayaux, Charles-Edouard Luyt, Alexandre Boissonnas, Zahir Amoura, Valérie Pourcher, Makoto Miyara, Guy Gorochov, Amélie Guihot, Christophe Combadière

**Affiliations:** ^1^Sorbonne Université, Institut national de santé et de recherche medicale (Inserm), Centre National de la Recherche Scientifique (CNRS), Centre d’Immunologie et des Maladies Infectieuses, Cimi-Paris, Paris, France; ^2^Assistance Publique – Hôpitaux de Paris (AP-HP), Groupement Hospitalier Pitié-Salpêtrière, Département d’Immunologie, Paris, France; ^3^Assistance Publique – Hôpitaux de Paris (AP-HP), Groupement Hospitalier Pitié-Salpêtrière, Service de Pneumologie, Médecine Intensive et Réanimation, Paris, France; ^4^Service de Médecine Intensive Réanimation, Institut de Cardiologie, Assistance Publique – Hôpitaux de Paris (AP-HP), Sorbonne Université, Hôpital Pitié – Salpêtrière, Paris, France; ^5^Sorbonne Université, Inserm, Institute of Cardiometabolism and Nutrition (ICAN), Paris, France; ^6^Service de Médecine Interne 2, Institut E3M, Assistance Publique – Hôpitaux de Paris (AP-HP), Hôpital Pitié-Salpêtrière, Paris, France; ^7^Assistance Publique – Hôpitaux de Paris (AP-HP), Groupement Hospitalier Pitié-Salpêtrière, Service de Maladies infectieuses et Tropicales, Paris, France

**Keywords:** neutrophils, COVID-19, infectious diseases, thrombosis, innate immunity

## Abstract

**Background:**

Lymphopenia and the neutrophil/lymphocyte ratio may have prognostic value in COVID-19 severity.

**Objective:**

We investigated neutrophil subsets and functions in blood and bronchoalveolar lavage (BAL) of COVID-19 patients on the basis of patients’ clinical characteristics.

**Methods:**

We used a multiparametric cytometry profiling based to mature and immature neutrophil markers in 146 critical or severe COVID-19 patients.

**Results:**

The Discovery study (38 patients, first pandemic wave) showed that 80% of Intensive Care Unit (ICU) patients develop strong myelemia with CD10^−^CD64^+^ immature neutrophils (ImNs). Cellular profiling revealed three distinct neutrophil subsets expressing either the lectin‐like oxidized low‐density lipoprotein receptor‐1 (LOX‐1), the interleukin-3 receptor alpha (CD123), or programmed death-ligand 1 (PD-L1) overrepresented in ICU patients compared to non-ICU patients. The proportion of LOX-1- or CD123-expressing ImNs is positively correlated with clinical severity, cytokine storm (IL-1β, IL-6, IL-8, TNFα), acute respiratory distress syndrome (ARDS), and thrombosis. BALs of patients with ARDS were highly enriched in LOX-1-expressing ImN subsets and in antimicrobial neutrophil factors. A validation study (118 patients, second pandemic wave) confirmed and strengthened the association of the proportion of ImN subsets with disease severity, invasive ventilation, and death. Only high proportions of LOX-1-expressing ImNs remained strongly associated with a high risk of severe thrombosis independently of the plasma antimicrobial neutrophil factors, suggesting an independent association of ImN markers with their functions.

**Conclusion:**

LOX-1-expressing ImNs may help identifying COVID-19 patients at high risk of severity and thrombosis complications.

## Introduction

Since the first reports of an outbreak of a severe acute respiratory syndrome caused by coronavirus 2 (SARS-CoV-2) in China in December 2019 (1, 2), the coronavirus disease (COVID-19) has grown to be a global public health emergency, with cases of COVID-19 around the world reaching 5.5 million and deaths from the disease standing at more than 90 000 as of May 2021 (for up-to-date data, see https://www.who.int/emergencies/diseases/novel-coronavirus-2019/situation-reports). The SARS-CoV-2 infection is characterized by a range of symptoms including fever, cough, fatigue, and myalgia in the majority of cases, and occasional headache and diarrhea (1, 3). Among reported cases, approximatively 80% present with a mild condition, 13% with a serious condition, and 6% in a critical state requiring intensive care; the latter is associated with a fatality rate of 2–8% of reported cases (4). Some severe cases of COVID-19 progress to acute respiratory distress syndrome (ARDS), which accounts for high mortality related to damage of the alveolar lumen. Numerous patients with ARDS secondary to COVID-19 develop life-threatening thrombotic complications (5).

Previous coronavirus-related infections have been characterized by the onset of a cytokine storm (6). The inflammatory response measured at both cellular and molecular levels could represent a strong prognostic signature of the disease. The cytokine storm is an uncontrollable inflammatory response leading to viral sepsis, ARDS, respiratory failure, shock, organ failure, or death (7, 8). Strong predictive markers are still missing for these complications.

Older age, neutrophilia, and organ and coagulation dysfunction are the major risk factors associated with the development of ARDS and progression to death (9). In addition, serum concentrations of both pro- and anti-inflammatory cytokines, including IL-6, TNFα, and IL-10, were increased in the majority of severe cases and were markedly higher than those of moderate cases, suggesting that the cytokine storm might be associated with disease severity (10, 11) and leading the way to the development of potential immune-modulatory treatments (3, 12). The cytokine storm is associated with a massive influx of innate immune cells, namely neutrophils and monocytes, which could worsen lung injury. The accumulation of innate effectors, in particular neutrophils, in the bronchoalveolar lavage (BAL) of COVID-19 patients correlates with pro-inflammatory cytokines and reflects the outcome of patients (13). However, our knowledge of a particular innate immune actor (i.e., neutrophils and molecular mechanisms) during severe COVID-19 is incomplete.

Increasing clinical data indicate that the neutrophil/lymphocyte ratio (NLR) is a powerful predictive and prognostic indicator of severe COVID-19 (14–16). Lymphopenia, neutrophilia, and high NLR are associated with a more severe viral infection (14, 17). It is well recognized that, in an emergency response to severe infection, the bone marrow releases mature and immature neutrophils (ImNs). ImNs have been shown to be enriched in granule antimicrobial, cytotoxic, and neutrophil extracellular trap (NET)-forming proteins (18). More recently, NETs were shown to be associated with COVID-19 severity and microthrombi formation (19–21).

We previously identified two new CD10−CD64+ neutrophil subsets, expressing either PD-L1 or CD123, that were specific to bacterial sepsis (22). In addition to these markers, previous work showed that LOX-1 is an important mediator of inflammation and neutrophil dysfunction in sepsis and cancers (23, 24). To test the hypothesis of a virally driven neutrophil profile, we developed a multiparametric neutrophil profiling strategy based on known neutrophil markers to distinguish COVID-19 phenotypes in patients in critical or severe condition.

## Materials and Methods

### Study Participants

Fresh blood samples from 38 (pilot study, Mar–Apr 2020) and 118 (validation study, Sep–Nov 2020) consecutive adult patients with COVID-19 referred to the Department of Internal Medicine 2, Department of Infectious Diseases and Intensive Care Units of Pitié-Salpêtrière University Hospital, Paris, were included. The diagnosis of COVID-19 relied on SARS-CoV-2 carriage on the nasopharyngeal swab, as confirmed by real-time reverse transcription PCR analysis. In addition, blood samples were collected from healthy donors obtained from the French blood donation center. When ventilator-associated pneumonia was suspected, patients underwent fiber-optic bronchoscopy and BAL (*n* = 16) to sample distal respiratory secretions. Leukocyte phenotyping was performed on BAL when possible. Demographic and clinical characteristics are detailed in **Tables 1** and **2**, respectively.

**Table 1:** Demographics and baseline characteristics of patients with COVID-19: pilot study.

	All patients (*N = *38)	ICU patients (*N = *24)	Non-ICU patients (*N = *14)
Men	25 (65.8)	18 (75)	7 (50)
Age, years, median (range)	57 (25–79)	55 (25–75)	65 (27–79)
**Chronic medical illness**			
Heart disease	4 (10.5)	4 (16.7)	0(0)
Type 2 diabetes	13 (34.2)	9 (37.5)	4 (28.6)
*Body mass index (kg/m^2^)*			
Normal (18.5–25)	19 (49.7)	9 (37.5)	9 (64.3)
Overweight (25–30)	5 (13.2)	3 (12.5)	3 (21.4)
Obesity (≥30)	14 (36.8)	12 (50)	2 (14.3)
Hypertension	19 (50)	11 (45.8)	8 (57.1)
Immunocompromised*	2 (5.3)	1 (4.2)	1 (7.1)
Malignant tumor	6 (15.8)	3 (12.5)	3 (21.4)
Chronic neurologic disease	1 (2.6)	1 (4.2)	0 (0)
Chronic pulmonary disease	5 (13.2)	4 (16.7)	1 (7.1)
Chronic kidney disease	6 (15.8)	3 (12.5)	3 (21.4)
Chronic liver disease	0 (0)	0 (0)	0 (0)
*Smoking habits*			
Never smoked	31 (81.6)	21 (87.5)	10 (71.4)
Former smoker	4 (10.5)	3 (12.5)	1 (7.1)
Daily smoker	3 (7.9)	0 (0)	3 (21.4)
*Past history of arterial or venous thrombosis*	6 (15.8)	4 (16.7)	2 (14.3)
Arterial	5 (13.2)	4 (16.7)	1 (7.1)
Venous	1 (2.6)	0 (0)	1 (7.1)
**Treatment regimen at baseline**			
Long-term immunosuppressive agent	1 (2.6)	1 (4.1)	0 (0)
Nonsteroidal anti-inflammatory drugs	0 (0)	0 (0)	0 (0)
Recent chemotherapy for cancer	2 (5.3)	1 (4.2)	1 (7.1)
Angiotensin converting enzyme inhibitor	10 (26.3)	6 (25)	4 (28.6)
Angiotensin II receptor blockers	6 (15.8)	4 (41.7)	4 (12.3)
Anticoagulant therapy	12 (31.6)	11 (45.8)	1 (7.1)
**Severity score at baseline**			
SAPS II, median (range)	33 (25–78)	35.5 (15–78)	25.5 (9–61)
SOFA, median (range)		8.5 (2–17)	
**Time from onset of symptoms to admission**			
Days, median (range)	8 (5–47)	8 (5–22)	13 (1–47)
**Laboratory findings at baseline**			
Leucocytes, ×10^9^/L, median (range) [normal range: 4.0–10.0]	9.29 (1.19–23.79)	10.3 (3.43–23.79)	7.705 (1.35–19.86)
Neutrophil count, ×10^9^/L, median (range) [normal range: 2.7–5]	7.87 (1.35–60.76)	8.75 (2.72–60.76)	7.705 (1.35–19.86)
Lymphocyte count, ×10^9^/L, median (quartiles) [normal range: 1.5–4]	0.94 (0.56–1.34)	0.925 (0.48–2)	1.185 (0.24–1.81)
Lactate dehydrogenase, U/L, median (range) [normal range: 135–215]	475.5 (234–2030)	504 (375–1087)	324 (234–2030)
D-dimers, ng/mL, median (range)	2450 (540–20 000)	2760 (540–20 000)	1860 (540–20 000)
**Chest CT finding: extension of GGO and/or consolidation** ^¤^			
0%	0 (0)	0 (0)	0 (0)
<10%	4 (14.3)	0 (0)	4 (33.3)
10–25%	4 (14.3)	1 (6.3)	3 (25)
25–50%	6 (21.4)	2 (12.5)	4 (33.3)
50–75%	9 (32.1)	8 (50)	1 (8.3)
>75%	5 (17.9)	5 (31.3)	0 (0)
**Treatment**			
*Hydroxychloroquine*	16 (42.1)	14 (58.3)	2 (14.3)
*Glucocorticoids*	1 (2.6)	1 (4.2)	0 (0)
*Tocilizumab or sarilumab*	0 (0)	0 (0)	0 (0)
*Oseltamivir*	5 (13.2)	5 (20.8)	0 (0)
*Antibiotic therapy*	38 (100)	24 (100)	14 (100)
*Oxygen therapy*	38 (100)	24 (100)	14 (100)
Nasal cannula	14 (36.8)	0 (0)	14 (100)
Noninvasive ventilation or high-flow nasal cannula	3 (7.9)	3	0
Invasive mechanical ventilation	21 (55.3)	21 (87.5)	0 (0)
*Extracorporeal membrane oxygenation*	13 (34.2)	13 (54.2)	0 (0)
*Hemodialysis*	10 (26.3)	8 (33.3)	2 (14.3)
**Complications**			
Acute respiratory distress syndrome	21 (55.3)	21 (87.5)	0 (0)
Acute kidney injury	12 (31.2)	11 (45.8)	1 (7.1)
Pulmonary embolism	2 (5.3)	1 (4.2)	1 (7.1)
Thrombosis	11 (28.9)	10 (41.7)	1 (7.1)
Venous	11 (28.0)	10 (41.7)	1 (7.1)
Arterial	0 (0)	0 (0)	0 (0)
**Clinical outcome^†^**			
Duration of hospitalization, days	17 (1–56)	20 (1–56)	17 (8–29)
Discharged	32 (84.2)	19 (79.2)	14 (100)
Remained in hospital	0 (0)	0 (0)	0 (0)
Death	5 (13.2)	5 (20.8)	0 (0)

*Including cardiac, liver or kidney allograft, hematopoietic stem cell transplantation, or immunosuppressive agent for autoimmune disease.

^¤^28 patients were assessed.

^†^As of December 2, 2020.

Values are expressed as n (%), unless stated otherwise. CT, computed tomography; GGO, ground-glass opacities; SAPS II, Simplified Acute Physiology Score II; SOFA, Sepsis Organ Failure Assessment.

**Table 2:** Correlation analyses between serum cytokine levels and marker-expressing neutrophil abundances.

Frequency of marker expression among neutrophils						
	CD123		LOX-1		PD-L1	
	*r*	*p-*value	*r*	*p-*value	*r*	*p-*value
SOFA	0.69	<0.0001****	0.68	0.0001***	0.55	0.0023**
D-dimers		ns	0.42	0.023*	0.37	0.039*
hIL1beta		ns	0.56	0.003**	0.39	0.036*
hIL6		ns	0.48	0.010*		ns
hIL8		ns	0.48	0.009		ns
hTNFa		ns	0.42	0.022*		ns
hIL10		ns	0.43	0.020*		ns
IL-17	0.06	0.035*		ns		ns
IL-18	0.22	0.009**		ns	0.48	0.0094**
hIL22	0.32	0.005**		ns	0.56	0.0027**
IFN-alpha		ns		ns	−0.53	0.0055
IFN-beta	−0.45	0.035*	−0.45	0.023*		ns
hIFN-gamma		ns		ns	0.53	0.0042**
GM-CSF		ns		ns	−0.40	0.03*
IL-3		ns	−0.44	0.020*		ns

Simoa (single molecule array) HD-1 analyzer was used for ultrasensitive multiplex immunodetection of cytokines as described in methods section. Potential association between serum cytokine levels and marker-expressing neutrophil frequencies was evaluated by Spearman’s correlation (one-tailed), with significance defined by a p-value < 0.05: *p < 0.05; **p < 0.01; ***p < 0.001; ****p < 0.0001; ns, not significant.

### Study Approval

The studies were conducted in accordance with the Declaration of Helsinki and the International Conference on Harmonization (ICH) Good Clinical Practice (GCP) Guideline and approved by the relevant regulatory and independent ethics committees. In accordance with current French law, informed written consent was obtained from patients or relatives. The studies were registered and approved by the local ethical committee of Sorbonne Université/Assistance Publique – Hôpitaux de Paris for standard hospitalized patients (N°2020-CER2020-21) and ICU patients (N° CER-2020-31).

### Flow Cytometry

One hundred microliters of fresh whole blood collected at admission to the hospital—on anticoagulant citrate-dextrose solution (ACD) for patients in the ICU or on EDTA for patients in standard hospitalization—was stained with a mix of monoclonal antibodies. Samples were diluted in Brilliant Violet buffer (BD Biosciences, Le Pont-de-Claix, France) and incubated 20 min at room temperature in the dark. The antibody panel included CD15-BV786, CD14-BUV737, and CD10-BUV395 (BD, Le Pont-de-Claix, France); and CRTH2-FITC, CD123-PE, LOX-1-BV421, CD64-BV605, and PD-L1-BV711 (BioLegend, San Diego, CA, USA) (**Supplementary Table S1**). One milliliter of BD FACS lysing solution 1X (BD Biosciences) was directly added to the cells to lyse red blood cells, which were incubated for 20 min, centrifuged, and washed with PBS. Leukocytes were resuspended in PBS before analysis with a BD LSRFortessa X-20 (BD Biosciences). FlowJo software 10.0 (FlowJo LLC, Ashland, OR, USA) was used for analysis of marker expression on neutrophils. One hundred microliters of whole blood were stained with a fluorescence minus one (FMO) mix missing antibodies targeting CD123, LOX-1, and PD-L1, in order to determine the threshold of expression of these markers. BAL leukocyte phenotyping was performed similarly after filtration, two wash procedures of BAL cells, and staining with the same antibody mix. Acquired data were normalized and analyzed using the OMIQ platform (https://www.omiq.ai). To identify neutrophil subsets and visualize all cells in a 2D map where position represents local phenotypic similarity, we used a dimensionality reduction tool: the opt-SNE implementation of t-SNE. Neutrophils (40 000 events) were randomly taken from the sample for the unsupervised analysis. Cells were also grouped in phenotypically homogeneous clusters using the FlowSOM algorithm.

### ELISA for Antimicrobial Proteins

MPO and neutrophil ELA were measured using Human Myeloperoxidase and Human Neutrophil Elastase/ELA2 DuoSet ELISA kits (R&D Systems, Minneapolis, MN, USA), with plasma or BAL diluted to 1:100 with PBS according to manufacturer’s instructions. The concentrations of MPO and ELA were expressed as picograms/milliliter or relative luminescence units. Netosis was measured in the patient’s plasma by detecting MPO-DNA complexes using anti-human MPO primary antibody (clone 4A4; AbD Serotec, Marnes-la-Coquette, France) as the capture antibody and a peroxidase-labeled anti-DNA antibody (clone MCA-33; Roche, Mannheim, Germany) as the detection antibody. Plasma samples were diluted 1:4 in PBS.

### Quanterix Technology (Digital ELISA)

The Simoa™ (single molecule array) HD-1 analyzer (Quanterix, Lexington, MA, USA) using singleplex bead-based assays was used for ultrasensitive immunodetection of IL-3, IL-17A, IL-18, GM-CSF, and IFN-α. Concentrations of IL-1β, IFN-g, IL-6, IL-8, IL-22, TNFα, and IL-10 were determined using a multiplex planar array immunoassay on the Quanterix SP-X platform according to manufacturer’s instructions. Serum IFN-β levels were quantified with a highly sensitive ELISA kit (PBL Assay Science, Piscataway, NJ, USA). The concentrations of cytokines in unknown samples were interpolated from a standard curve created with two replicates of each level of recombinant calibrator proteins representing the dynamic range of the assay: IL-1β (0.073–300 pg/mL), IFN-g (0.012–50 pg/mL), IL-6 (0.073–300 pg/mL), IL-8 (0.098–400 pg/mL), IL-22 (0.024–100 pg/mL), TNFα (0.098–400 pg/mL), IL-10 (0.024–100 pg/mL), IL-3 (0.686–500 pg/mL), IL-17A (0.041–30 pg/mL), IL-18 (0.011–45 pg/mL), GM-CSF (0.041–30 pg/mL), IFN-α (0.028–27.3 pg/mL), and IFN-β (1.2–150 pg/mL) (**Supplementary Table S1**).

### Data Presentation and Statistical Analysis

Statistical analyses of the immunological data and graphic representations were performed with Prism 9 (GraphPad Software Inc.). A nonparametric Mann–Whitney test was used for group comparisons, and one-way ANOVA tests with a Tukey’s adjustment was used for multiple group comparison tests. The association between variables was evaluated using Spearman’s correlation (one-tailed), with significance defined by a *p*-value of <0.05. Survival curves were compared with a log-rank (Mantel–Cox) test and were considered statistically significant with a *p* of <0.05. HR values with 95% CI were computed. ﻿ROC curves were created using Prism 9. PCAs were performed with R-software 3.3.1.

## Results

### Increased Proportions of Circulating Immature Neutrophils Expressing Either CD123 or LOX-1 in Critical COVID-19 Patients Are Associated With COVID-19 Severity and Thromboembolic Complications

We designed a first discovery study with 38 individuals and analyzed their neutrophil phenotypes, comparing them with those of patients admitted to the ICU (*n* = 24) or not (*n* = 14), within the first day following their admission to the ICU or hospitalization units (see **Table 1** and **Supplementary Results** for demographic and clinical characteristics). COVID-19 patients from ICUs displayed more severe clinical and biological signs than non-ICU patients, with an elevated Simplified Acute Physiology Score (SAPS) II (ICU: 35.5, *n* = 24 and non-ICU: 25.5, *n* = 14; *p-*value = 0.05), higher serum lactate dehydrogenase (ICU: 504, *n* = 24 and non-ICU: 324, *n* = 14; *p-*value = 0.005), and higher D-dimers (ICU: 2760, *n* = 23 and non-ICU: 1860, *n* = 12; *p-*value = 0.25). However, they did not differ in neutrophil counts or levels of lymphopenia (**Table 1**). Patients’ characteristics confirmed previously published data, with a notably high prevalence of obese COVID-19 patients. The main differences between ICU and non-ICU patients reflected case severity, with a high proportion of patients with lung lesions (observed as ground-glass opacities (GGOs) on chest CT scans) requiring invasive mechanical ventilation (IMV) and resulting in high in-hospital mortality.

**Figure 1:**
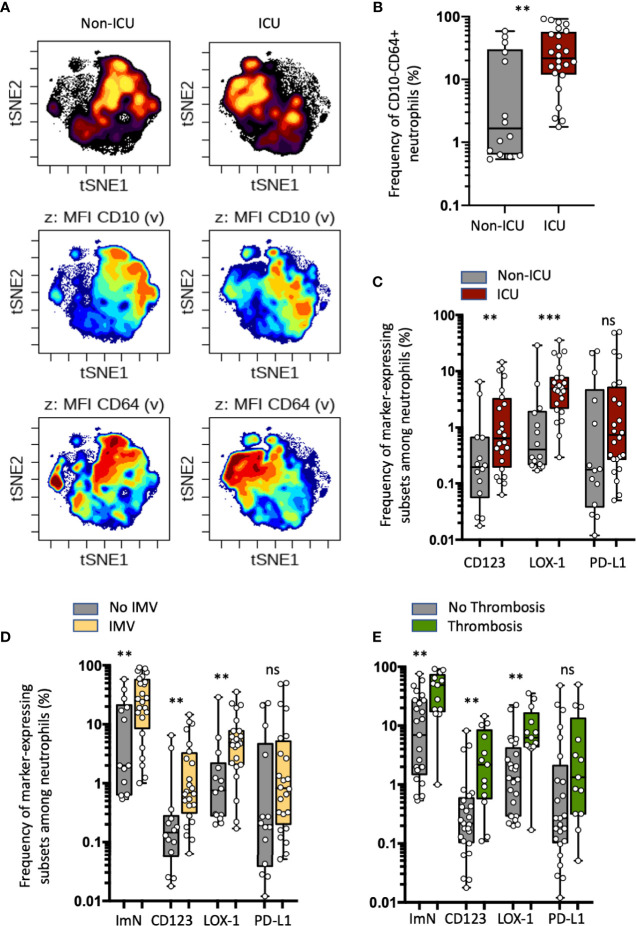
Increased proportions of circulating immature neutrophils expressing either CD123 or LOX-1 in critical COVID-19 patients are associated with COVID-19 severity and thromboembolic complications. **(A)** Opt-SNE analysis (OMIQ) was performed on 40 000 randomly chosen neutrophils from all samples with cells organized along t-SNE-1 and t-SNE-2 according to per-cell expression of CD15, CD10, CD64, LOX-1, CD123, and PD-L1. Cell density for the concatenated file of each patient’s group (ICU *vs* non-ICU patients from the discovery study) is shown on a black-to-yellow heat scale. Expression of neutrophil CD10 and CD64 markers is presented on a rainbow heat scale on the t-SNE map of each concatenated file. **(B)** Box plots (min to max distribution) of CD10^−^CD64^+^ neutrophil subset abundance among total neutrophils of each group’s samples. **(C)** Abundance of CD10^−^CD64^+^ neutrophils expressing CD123, PD-L1, or LOX-1 in ICU and non-ICU patient groups. **(D, E)** Box plots (min to max distribution) of the proportion of total ImNs and ImNs expressing CD123-, LOX-1, or PD-L1 in patient groups with invasive mechanical ventilation **(D)** or with thromboembolic complications E. Nonparametric Mann–Whitney test was used to compare differences in cellular abundance of neutrophil subsets between groups, with significance defined by a *p*-value < 0.05: **p < 0.01; ***p < 0.001 and ns, not significant.

Within 3 h of drawing blood, we performed whole blood immunostaining using a previously published panel designed to precisely evaluate immature circulating neutrophils (22). Neutrophils were automatically identified and visualized using a dimensionality reduction tool (opt-SNE for optimal implementation of t-distributed stochastic neighbor embedding (t-SNE)) to define an imprint for each sample group (**Figure 1A**). Under unsupervised classification, neutrophils from ICU patients were organized in the upper left quadrant of the map, whereas those from the non-ICU patients ended up in the upper right quadrant. This analysis allowed delimitation of two main subsets of neutrophils based on the expression of CD10 and CD64 markers: the ICU-abundant upper left area was composed of neutrophils with mid-to-low expression of CD10 and high expression of CD64, and the non-ICU-abundant upper right area was composed of high-to-mid expression of CD10 and high-to-mid expression of CD64. We next determined whether the identified neutrophil signature would be confirmed by conventional analysis undertaken by experts. Neutrophils were identified with the CD15 neutrophil marker, while excluding prototypical markers of eosinophils and monocytes, respectively CRTH2 and CD14 (see **Supplementary Figures S1A** and **S1B** for representative patient samples). An expert-gating strategy confirmed the high abundance of CD10^−^CD64^+^ ImNs among ICU patients compared with non-ICU patients (**Figure 1B**). We did not observe any correlation between ImN proportion and age, obesity, hypertension, diabetes, and smoking (data not shown). We next compared the expression of CD123, LOX-1, and PD-L1 surface molecules, formerly known to be dysregulated in sepsis (22). All three of them were barely co-expressed on neutrophils (**Supplementary Figure S1C**), which led to the identification of three distinct ImN subpopulations. Subsets of neutrophils expressing either CD123 or LOX-1 were more abundant in ICU than in non-ICU patients (**Figure 1C**), unlike those expressing PD-L1, for which overabundance did not reach statistical significance (*p* = 0.1). Principal component analysis (PCA) revealed that CD123-, LOX-1-, or PD-L1-expressing ImNs contributed independently to the disease severity of patients as evaluated by SAPS II (see **Supplementary Figure S2A**). In addition, PCA confirmed that the disease severity in ICU patients was associated with the expression of CD123, LOX-1, and PD-L1 on ImNs and distinct patterns of cytokines (see **Supplementary Figure S2B** and **Table 2**). We identified three profiles: (a) patients with high LOX-1-expressing ImN proportions and high IL-1β, IL-6, IL-8, and TNFα serum levels; (b) patients with CD123 and PD-L1 expression and IL-18, IL-22, and IFNγ secretion; and (c) patients with a lower severity score associated with high type-1 interferon levels. Thus, ImN subsets expressing either CD123, LOX-1, or PD-L1 may define specific profiles of severity associated with high levels of cytokines.

Because the mortality in COVID-19 cases is associated with the virally driven cytokine storm, especially in patients with comorbidities (1), we sought to correlate ImN subsets with severe symptoms such as ARDS (patients receiving intermittent mandatory ventilation (IMV)) or thrombosis (**Figures 1D, E**, respectively). The requirement for IMV or the occurrence of thromboembolic events was associated with higher proportions of ImN expressing CD123 or LOX-1, unlike those expressing PD-L1 (*p* = 0.1). No differences in the proportions of ImN subsets could be detected between discharged and deceased patients (see **Supplementary Figure S2C**). These data suggested that increased proportions of circulating ImNs expressing either CD123 or LOX-1 in critical COVID-19 patients are associated with COVID-19 severity and thromboembolic complications.

### Subsets of Immature Neutrophils Expressing LOX-1 Infiltrate Lung

**Figure 2:**
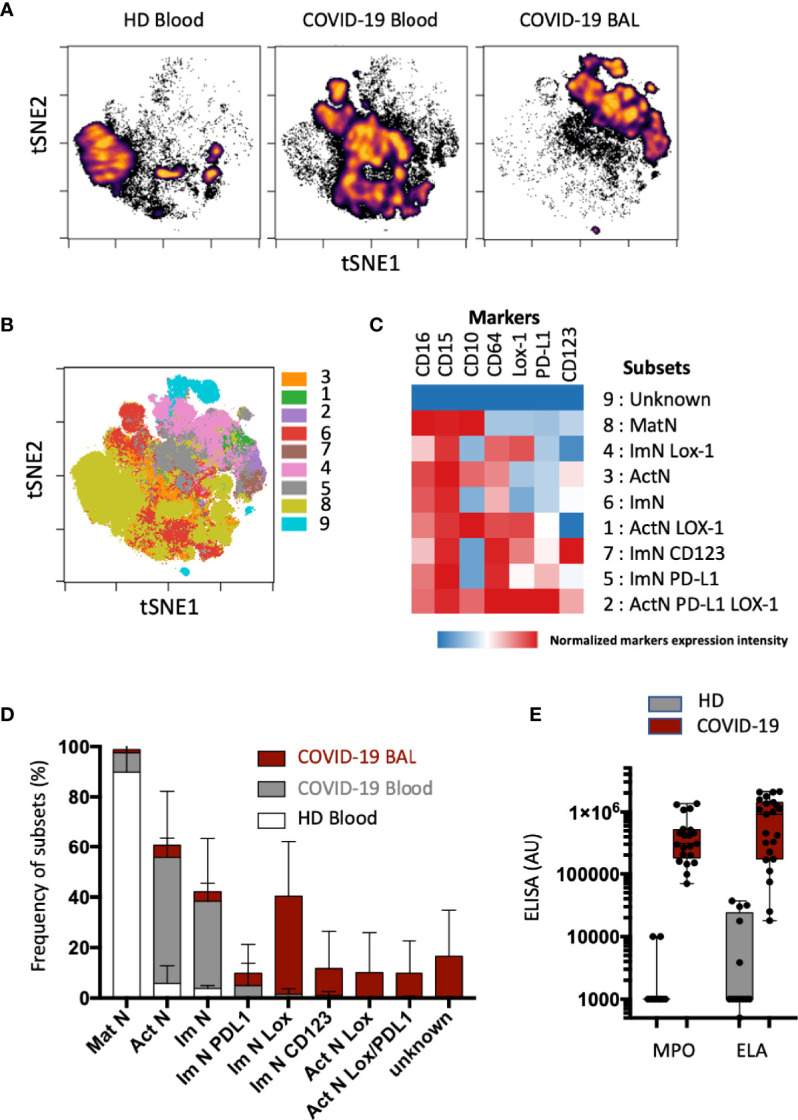
Subsets of immature neutrophils expressing LOX-1 infiltrate lung. **(A)** Opt-SNE analysis was performed on 40 000 randomly chosen neutrophils from blood (*n* = 16) and bronchoalveolar (BAL) samples (*n* = 16). Cells were organized along t-SNE-1 and t-SNE-2 according to per-cell expression of CD10, CD15, CD16, CD64, LOX-1, CD123, and PD-L1. Cell density for the concatenated file of each group; blood samples from healthy donor (HD) blood; *n* = 8); blood and BAL samples from COVID-19 patients (*n* = 16). Cell density is shown on a black-to-yellow heat scale. **(B)** Spatial t-SNE representing nine major clusters of neutrophils automatically arranged with FlosSom (OMIQ). **(C)** Heatmap representation of mean signal intensity of each marker in identified neutrophil subsets. Subsets were annotated according to the following markers: mature neutrophils (MatNs) expressed all three CD10, CD15, and CD16 markers; activated neutrophils (ActNs) expressed all four CD10, CD15, CD16, and CD64 markers; and ImNs expressed positive CD15, CD16, and CD64 but negative CD10. Heat intensity (from blue to red) reflects the normalized expression of each marker. **(D)** Box plots with SD of neutrophil subset abundances in HD blood (white bar); and COVID-19 blood (gray bar) and BAL (red bar). **(E)** Box and whisker plots with min and max of myeloperoxidase (MPO) and neutrophil elastase (ELA) in BAL of COVID-19 patients (*n* = 12) and of healthy donors obtained from the French blood donation center (HD; *n* = 12).

A pulmonary immune environment during critical COVID-19 infection is one of the major features of disease complications. We thus sought neutrophil subsets in the bronchoalveolar lavages (BALs) when available and compared them to blood samples from patients and healthy donors (**Figure 2**). With a opt-SNE algorithm, BAL neutrophils were identified in the upper right quadrant of the map; blood neutrophils from COVID-19 patients were more central; and blood neutrophils from healthy donors (HD) were organized in the lower left quadrant of the map (**Figure 2A**). Automatic clustering using major (CD15, CD10, CD16, CD64) and specific (CD123, LOX-1, PD-L1) neutrophil markers split neutrophil signatures into positive and negative subpopulations for each marker (**Figure 2B** and **Supplementary Figure S3**). This unsupervised analysis allowed the identification of nine clusters, representing three main subsets of neutrophils (**Figure 2C**): (a) the mature neutrophils (MatN or subset 8) with high expression of CD15 and CD10 and low expression of CD64; (b) the ImNs with high expression of CD15 and CD64 and low expression of CD10(ImN or subset 6); and (c) the activated neutrophils (ActN or subset 3) with high expression of CD15, CD10, and CD64. Expression of LOX-1 and PD-L1 was spotted on ActN (subset 1 and 2) whereas LOX-1, CD123, and PD-L1 expression was observed on three independent ImN subsets (subsets 4,7 and 5). MatNs were abundant in healthy donor blood, and both ActNs and ImNs were abundant in the blood of COVID-19 patients (**Figure 2D**). If ImNs expressing LOX-1, PD-L1, or CD123 represented a few percent of COVID-19 blood neutrophils (**Figure 2D**; see also **Figure 1C**), these subsets were much more present in patient BALs with ImNs expressing LOX-1 being the major subset, representing about 40% of total neutrophils. A profusion of ImNs in COVID-19 BAL was associated with massive production of myeloperoxidase (MPO) and neutrophil elastase (ELA) (**Figure 2E**), two antimicrobial and cytotoxic proteins known to be highly concentrated in the azurophilic granule of ImN.

These data revealed that ImNs, preferentially those expressing LOX-1, infiltrate bronchoalveolar space in the lungs of COVID-19 patients, where they release their cytotoxic content, suggesting a potential role in disease severity.

### Immature Neutrophil Subsets Expressing CD123, LOX-1, or PD-L1 Are Correlated With Clinical Severity, but Only the LOX-1+ Subset Proportion at Entry Is Strongly Associated With Higher Risk of Thrombosis

**Figure 3:**
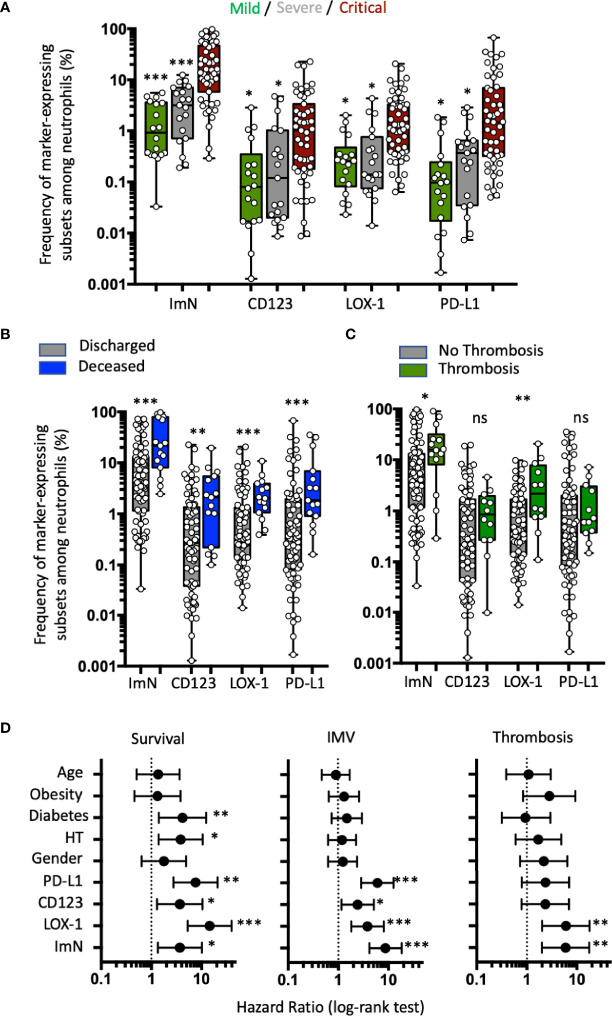
Immature neutrophil subsets expressing either CD123, LOX-1, or PD-L1 are correlated with clinical severity, but only LOX-1+ subset proportion at entry is strongly associated with higher risk of thrombosis. **(A)** Box plots (min to max distribution) of the proportion of ImNs expressing CD123-, LOX-1, or PD-L1 in severity patient groups in mild (*n* = 18), severe (*n* = 19), or critical (*n* = 51) clinical condition observed in patients from the validation study. One-way ANOVA with a Tukey’s adjustment was used to compare the three groups (critical *vs* severe or critical *vs* mild), with significance defined as follows: *p < 0.05; and ***p < 0.001. **(B)** Box plots (min to max distribution) of the abundance of CD10−CD64+ neutrophil subsets among discharged (*n* = 74) and deceased (*n* = 14) patients. Nonparametric Mann–Whitney test was used to compare differences between groups, with significance defined by a *p*-value < 0.05: * **p < 0.01; ***p < 0.001; ns, not significant. **(C)** Box plots (min to max distribution) of the abundance of CD10−CD64+ neutrophil subsets among patients without (*n* = 75) or with (*n* = 12) thromboembolic complications. Nonparametric Mann–Whitney test was used to compare differences in cellular abundance of neutrophil subsets between groups, with significance defined by a *p*-value < 0.05: *p < 0.05; **p < 0.01. **(D)** Forest plots comparing hazard ratio (log-rank test) for survival, invasive mechanical ventilation (IMV), and thrombotic events (thrombosis) in 118 patients according to median frequency of ImN subsets (reference group with ImN subset frequencies below the median) and comorbidities (reference group with no comorbidities or age below 61). Log-rank (Mantel–Cox) test was used to compare HR between groups, with significance defined by a *p*-value < 0.05: *p < 0.05; **p < 0.01; and ***p < 0.001.

COVID-19 patients from the validation study were segregated into three groups based on severity of disease at the time of admission: 18 in mild, 19 in severe, and 51 in critical condition (**Table 3** and **Supplementary Results**). The proportion of CD123-, LOX-1-, and PD-L1-expressing ImNs correlated positively with severity (**Figure 3A**). Interestingly, abundances of all three ImN subsets were associated with patient death (**Figure 3B**) and with patients requiring IMV (see **Supplementary Figure S4**), but only the LOX-1-expressing ImN subset was associated with thromboembolic events (**Figure 3C**), confirming our results in the pilot study (see **Figure 1E**). We segregated our COVID-19 patients into two groups based on the median proportion of each ImN subset and compared their relative risk of ARDS, thrombosis, and death using a Cox proportional hazards model with other variables (age, gender, hypertension, obesity, and diabetes) (**Figure 3D**). Patients with a high abundance of ImN subsets were at higher risk of ARDS requiring IMV and of death. Hypertension and diabetes were risk factors of survival but not for ARDS. Again, only patients with a high abundance of LOX-1-expressing ImNs were at higher risk of thromboembolic complications (HR, 5.99; 95% CI, 2.02–17.81; *p* = 0.007).

**Table 3:** Demographics and baseline characteristics of patients with COVID-19: validation study.

	All patients (*N=*118)	ICU patients (*N = *69)	Non-ICU patients (*N = *49)
Men	75 (63.6)	43 (62.3)	32 (65.3)
Age, years, median (range)	61 (21–89)	61 (21–85)	63 (32–89)
**Chronic medical illness**			
Heart disease	23 (19.5)	12 (17.4)	11 (22.5)
Type 2 diabetes	36 (30.5)	26 (37.7)	10 (20.4)
*Body mass index (kg/m^2^)*			
Normal (18.5–25)	49 (41.5)	9 (37.5)	(64.3)
Overweight (25–30)	34 (28.8)	22 (32.4)	12 (24.5)
Obesity (≥30)	37 (31.4)	25 (36.8)	12 (24.5)
Hypertension	62 (52.5)	39 (56.5)	23 (46.9)
Immunocompromised*	12 (10.2)	7 (10.1)	5 (10.2)
Malignant tumor	11 (9.3)	7 (10.1)	4 (8.2)
Chronic neurologic disease	6 (5.1)	1 (1.5)	5 (10.2)
Chronic pulmonary disease	25 (21.2)	14 (20.3)	11 (22.5)
Chronic kidney disease	20 (17)	10 (14.5)	10 (20.4)
Chronic liver disease	5 (4.2)	2 (2.9)	3 (6.1)
*Smoking habits*			
Never smoked	85 (72)	52 (75.4)	32 (65.3)
Former smoker	30 (25.4)	15 (21.7)	15 (30.6)
Daily smoker	4 (3.4)	2 (2.9)	2 (4.1)
*Past history of arterial or venous thrombosis*	16 (13.6)	10 (14.5)	6 (12.2)
Arterial	9 (7.6)	4 (5.8)	5 (10.2)
Venous	9 (7.6)	8 (11.6)	1 (2)
**Treatment regimen at baseline**			
Long-term immunosuppressive agent	16 (13.6)	9 (13.0)	7 (14.3)
Recent chemotherapy for cancer	1 (0.9)	1 (1.5)	0 (0)
Angiotensin converting enzyme inhibitor	13 (11)	8 (11.6)	5 (10.2)
Angiotensin II receptor blockers	25 (21.2)	18 (26.1)	7 (14.3)
Anticoagulant therapy	15 (12.7)	9 (13.0)	6 (12.2)
**Severity score at baseline**			
SAPS II, median (range)	25 (6–88)	27.5 (6–88)	18 (6–43)
**Time from onset of symptoms to admission**			
Days, median (range)	7 (0–18)	7 (0–18)	5 (0–18)
**Laboratory findings at baseline**			
Leucocytes, ×10^9^/L, median (range) [normal range: 4.0–10.0]	7.55 (1.54–43.45)	9.03 (2.65–43.45)	5.69 (1.54–17.39)
Neutrophil count, ×10^9^/L, median (range) [normal range: 2.7–5]	6.05 (1.00–33.89)	7.02 (1.75–33.89)	3.94 (1.00–15.49)
Lymphocyte count, ×10^9^/L, median (quartiles) [normal range: 1.5–4]	0.93 (0.00–4.78)	0.78 (0–4.78)	1.08 (0.27–2.77)
Lactate dehydrogenase, U/L, median (range) [normal range: 135–215]	368 (184–999)	431 (184–999)	350 (189–586)
D-dimers, ng/mL, median (range)	1120 (240–20 000)	1520 (240–20 000)	720 (240–20 000)
**Chest CT finding: extension of GGO and/or consolidation** ^¤^			
0%	5 (0)	2 (2.9)	3 (6.1)
<10%	11 (9.3)	4 (5.8)	7 (14.3)
10–25%	32 (27.1)	9 (13.0)	23 (46.9)
25–50%	27 (22.9)	20 (29.0)	7 (14.3)
50–75%	22 (18.6)	21 (30.4)	1 (2.0)
>75%	6 (5.1)	6 (8.7)	0 (0)
**Treatment**			
*Hydroxychloroquine*	0 (0)	0 (0)	0 (0)
*Glucocorticoids*	90 (76.3)	59 (85.5)	31 (63.3)
*Tocilizumab or sarilumab*	5 (4.2)	2 (2.9)	3 (6.1)
*Oseltamivir*	0 (0)	0 (0)	0 (0)
*Antibiotic therapy*	35 (29.7)	30 (43.5)	5 (10.2)
*Oxygen therapy*	106 (89.8)	69 (100)	37 (75.5)
Nasal cannula	42 (35.6)	9 (13.0)	37 (75.5)
Noninvasive ventilation or high-flow nasal cannula	26 (22.0)	24 (34.8)	0 (0)
Invasive mechanical ventilation	38 (32.2)	36 (52.2)	0 (0)
*Extracorporeal membrane oxygenation*	19 (16.1)	19 (27.5)	0 (0)
*Hemodialysis*	13 (11.0)	10 (14.5)	3 (6.1)
**Complications**			
Acute respiratory distress syndrome	38 (32.2)	38 (55.1)	0 (0)
Pulmonary embolism	7 (5.9)	5 (7.2)	2 (4.1)
Thrombosis	6 (5.1)	6 (8.7)	0 (0)
Venous	6 (5.1)	6 (8.7)	0 (0)
Arterial	0 (0)	0 (0)	0 (0)
**Clinical outcome^†^**			
Duration of hospitalization, days	14 (1–78)	20 (2–78)	8 (1–33)
Discharged	93 (78.8)	47 (68.1)	46 (93.9)
Remained in hospital	5 (4.2)	5 (7.2)	0 (0)
Death	20 (17)	17 (24.6)	3 (6.1)

*Including cardiac, liver or kidney allograft, hematopoietic stem cell transplantation, or immunosuppressive agent for autoimmune disease.

^¤^103 patients were assessed.

^†^As of December 2, 2020.

Values expressed as n (%), unless stated otherwise. CT, computed tomography; GGO, ground-glass opacities; SAPS II, Simplified Acute Physiology Score II; SOFA, Sepsis Organ Failure Assessment.

These data from the validation study confirmed that ImN subsets expressing either CD123, PD-L1, or LOX-1 were associated with COVID-19 severity, but only LOX-1 expression remained associated with thromboembolic complications.

### Immature Neutrophil Subsets and Plasma Levels of MPO and ELA Are Independent Markers of COVID-19 Severity

**Figure 4:**
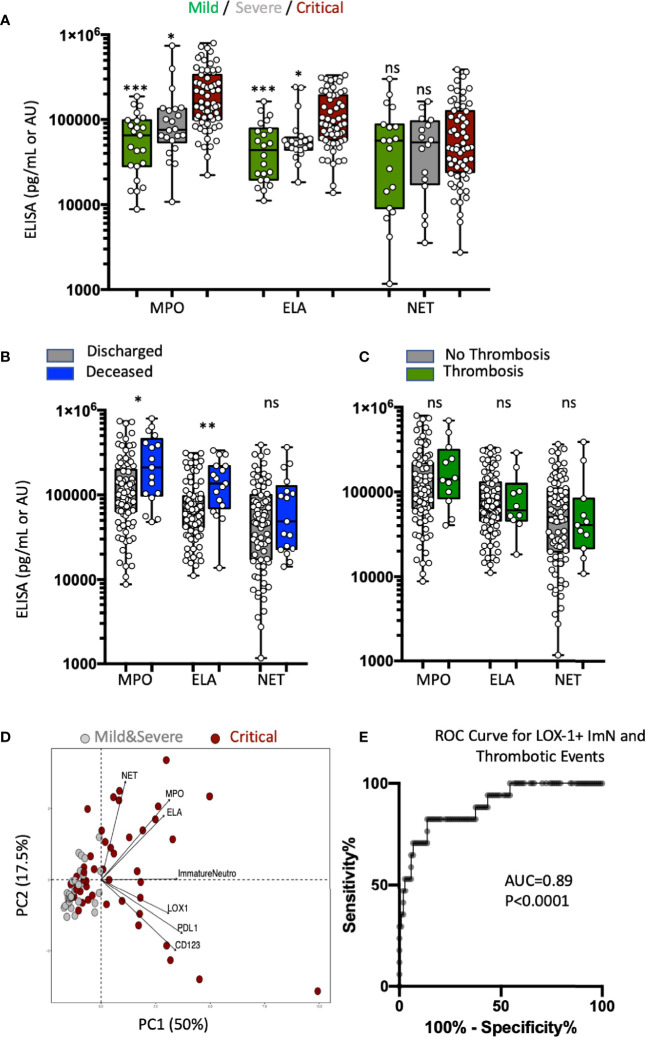
Immature neutrophil subsets and plasma levels of MPO and ELA are independent markers of COVID-19 severity. **(A)** Box plots (min to max distribution) of the plasma levels of MPO, ELA, and MPO-DNA complexes (NET) in severity patient groups in mild (*n* = 23), severe (*n* = 22), or critical (*n* = 63) clinical condition observed in patients from the validation study. The concentrations of MPO are expressed as pg/mL, ELA as 10^−1^ pg/mL, and MPO-DNA complexes as an arbitrary unit proportional to 10^−5^ of the ratio blank/sample of the absorbance measured at 450 nm. One-way ANOVA with a Tukey’s adjustment was used to compare the three groups (critical *vs* severe or critical *vs* mild), with significance defined as follows: *p < 0.05; and ***p < 0.001. **(B)** Box plots (min to max distribution) of the plasma levels of MPO, ELA, and NET among discharged (*n* = 90) and deceased (*n* = 17) patients. Nonparametric Mann–Whitney test was used to compare differences in cellular abundance of neutrophil subsets between groups, with significance defined by a *p*-value < 0.05: *p < 0.05; and **p < 0.01. **(C)** Box plots (min to max distribution) of the plasma levels of MPO, ELA, and NET among patients without (*n* = 94) or with (*n* = 12) thromboembolic complications. Nonparametric Mann–Whitney test was used to compare differences in cellular abundance of neutrophil subsets between groups, with significance defined by a *p*-value < 0.05: ns, not significant. **(D)** Principal component analysis (PCA) using granule protein plasma levels (MPO, ELA, and NET), immature phenotypic markers (LOX-1^+^, PD-L1^+^, CD123^+^ ImNs) and severity variables: critical patients, *n* = 63 (dark red circles); mild + severe patients, *n* = 45 (gray circles). **(E)** Receiver operating characteristic (ROC) curve analysis performed on combined discovery and validation studies to assess the predictive value of LOX-1 with thrombosis (*n* = 118).

Because ImNs that recently emigrated from the bone marrow are enriched in granule antimicrobial, cytotoxic, and NET-forming proteins, we measured plasma levels of MPO, ELA, and MPO-DNA complexes representing NET formation in three groups of patients on the basis of severity at the time of admission: 23 in mild, 22 in severe, and 63 in critical condition. MPO and ELA plasma levels were significantly associated with disease severity (**Figure 4A**), whereas ELA-DNA complexes were not. In addition, MPO and ELA levels at hospital admission were also significantly increased among COVID-19 patients who later died (**Figure 4B**). MPO-DNA complex levels were not associated with survival. There was no association between MPO, ELA, or MPO-DNA complexes and thromboembolic events (**Figure 4C**). PCA (**Figure 4D**) that combined abundances of ImN subsets and plasma levels of neutrophil microbicidal proteins revealed at least two independent patient profiles at risk of severity: those with high proportions of ImN subsets and those with high plasma levels of MPO, ELA, and NET. To further evaluate the ability of LOX-1 neutrophil markers to segregate patients with thrombosis, we plotted a receiver operating characteristic (ROC) curve (**Figure 4E**). The ROC analysis of these abundances indicated the optimal threshold yielding the best separation of the two groups of patients with optimal sensitivity and specificity. The AUC was 0.89 for LOX-1 ImN abundance (*p* < 0.0001), indicating that LOX-1 expression on ImN in the blood at the time of hospital admission could accurately predict later thromboembolic events among COVID-19 patients during hospitalization. A cutoff point of 0.5% abundance of the LOX-1 ImN subset was able to detect patients with thrombotic events with a sensitivity of 100% and patients without complications with a specificity of 53%. A cutoff point of 2% reached a sensitivity of 82% and a specificity of 75%.

## Discussion

Stratification of patients by using biomarkers remains an unmet need in COVID-19 patient care. Here, we sought to identify innate immune cellular signatures that may help predict the outcome of COVID-19 patients with severe symptoms. Conventional whole blood flow cytometry identified classical hallmarks of severe infection, such as neutrophilia and myelemia, but also revealed three novel neutrophil subsets able to distinguish between patients requiring ICU and those who do not. All three immature CD10^−^CD64^+^ neutrophil subsets expressing either CD123, LOX-1, or CD123 strongly correlated with severity scores commonly used in clinical practice: SAPS II and the Sequential Organ Failure Assessment (SOFA). They were also associated with distinct cytokine profiles. We found that high blood proportions of LOX-1+ neutrophils could be an important predictive signature of thromboembolic events, while D-dimer levels, an important negative predictor of thrombosis, were found to be high in a vast majority of patients. The strength of this study is that the main results were observed in the pilot study and confirmed in the validation study, representing samples from more than 150 patients collected over the course of two independent pandemic waves in France.

Previously, we reported that the proportion of CD123-expressing ImNs was correlated with bacterial sepsis severity (22). Here, we showed that the expression of CD123 on CD10^−^CD64^+^ neutrophils was associated with COVID-19 severity (higher SOFA or SAPS II scores, longer length of stay, and higher risk of death). Our results also suggested that both CD123 and its cognate ligand, the IL-3 cytokine, play an important role in sepsis. High IL-3 plasma levels have been associated with lung inflammation, lung injury, and high mortality rates in an animal model, as well as in humans (25, 26). IL-3 neutralization and anti-CD123 treatment improved mice outcome by decreasing inflammation and mortality rates (25). IL-3 promotes emergency myelopoiesis, exacerbating pro-inflammatory cytokine secretion and, consequently, systemic inflammation, organ dysfunction, and death. However, we did not observe similar results in COVID-19, as we reported an inverse correlation between IL-3 levels and SOFA scores. The association between CD123 expression on ImN and high serum levels of IL-17, IL-22, and IFNγ, to the best of our knowledge, has never been reported and may reveal a yet unidentified link between innate and adaptive immune responses. These findings open the way to new therapeutic opportunities aiming to control the excessive inflammation induced by SARS-CoV-2 infection.

Single-cell technology approaches to assessing dysregulation in the myeloid compartment have identified subsets of dysfunctional neutrophils in COVID-19 patients (27, 28). PD-L1-expressing neutrophils were detected only in patients with severe COVID-19. The authors revealed that this subset displayed surface markers and gene expression profiles reminiscent of myeloid-derived suppressor cells (MDSCs), suggesting that they may be involved in immune regulation by suppressing other immune cell activity. PD-L1-expressing MDSCs were previously described in HIV (29) and in cancer patients (30); they were also suggested to be involved in the immunosuppressive activity of T cell functions. Whether these PD-L1 neutrophils are dysfunctional or ImNs remains unclear. Emergency granulopoiesis in response to infections is well-documented (31). It mobilizes ImNs equipped with stocks of innate defense armory that are packaged into different granule subsets (32). ProNETosis gene signatures were observed in both pro- (*MPO*, *ELA*, and *PRTN3*) and pre-(*PADI4*) neutrophils and were associated with COVID-19 severity. Other reports have shown that NETosis increased in COVID-19 patients with ARDS and thrombotic complications (33, 34). We measured high levels of MPO and ELA in patients with critical conditions, but we did not observe any association between NETosis and patients’ clinical characteristics in the validation study. A potential bias of our study is that patients’ samples were obtained on the very first day of hospital admission and NETosis may have only been at its early stages. Active anticoagulant therapy administered to every patient may have prevented it (35).

LOX-1 was originally discovered as a receptor for oxLDL (36). oxLDL binding to LOX-1 results in oxLDL internalization and cellular accumulation, which triggers intracellular signaling processes leading to pro-apoptotic, pro-oxidant, and pro-inflammatory pathways, causing cell dysfunction associated with atherosclerosis and increased cardiovascular disease (CVD) risk (37). Binding of endothelial LOX-1 by ligands induces superoxide generation, inhibits nitric oxide production, enhances endothelial adhesiveness for leukocytes, and induces expression of chemokines (38).

In addition, LOX-1 binds other ligands with poor or no structural similarities to lipoproteins (for review (39) including phospholipids, proteins, bacteria and cells. It may have worked as host-defense molecule and still maintains some amino acid sequence homology with C-type lectin receptor.

LOX-1 expression and functions on neutrophils remain elusive. LOX-1 is barely detected on neutrophils during homeostasis, while its expression increases on neutrophils from human cancer patients (23) and in murine sepsis (24, 40). LOX-1 deletion in a murine model of polymicrobial sepsis reduced IL-6 and TNFα levels in the blood and lungs, which enhanced bacterial clearance and prevented neutrophil activation (24). LOX-1 was identified as a marker on granulocytic MDSCs able to suppress T cell activity (23) and produced high amount ROS. Recently, the work of Cabrera et al. revealed that ImN of severe COVID-19 patients had significantly more LOX-1+ cells as compared to mild cases and healthy controls (41) but T cell suppressive activity could not be evaluated. However, LOX-1 is mostly acknowledged for its role in atherosclerosis. LOX-1 is a class E scavenger receptor contributing to the formation of atherosclerotic plaques by promoting endothelial cell activation, macrophage foam cell formation, and smooth muscle cell migration and proliferation. LOX-1 activation induces NF-κB activation, leading to pro-inflammatory cytokine release, endoplasmic reticulum stress, and ROS production, which could damage the microenvironment (38, 39).

In this study, LOX-1 expression on ImNs seems to be detrimental for patients, as it was associated with the secretion of several pro-inflammatory cytokines, such as IL-6, IL-1β, and TNFα, with severity and a higher risk of thrombosis. In severe cases of COVID-19, the integrity of the lung is compromised by an exaggerated immune response leading to ARDS (17, 42). Mechanisms contributing to microcirculation disorders in sepsis are capillary leakage, leukocyte adhesion and infiltration, and intravascular coagulation, leading to thrombus formation. Over the course of systemic inflammatory diseases such as sepsis, the microenvironment is highly oxidative, leading to an increase of oxidized low-density lipoprotein (oxLDL) in plasma, which triggers LOX-1 overexpression through a positive feedback loop. In physiological conditions, the increase of LOX-1 expression, especially by endothelial cells, leads to an increase of LDL uptake by vessel walls, which activates the specific Oct-1/SIRT1 thrombosis protective pathway (43, 44). In this study, we observed an increase in vascular thrombotic events among individuals displaying a high frequency of immature LOX-1+ neutrophils. Whether thrombosis in COVID-19 patients results from functionally diverted neutrophils expressing LOX-1 or from its expression on endothelial and smooth muscle cells remains to be elucidated. We also observed a slight correlation between LOX-1-expressing ImNs and D-dimer levels (Spearman’s correlation, *r* = 0.42 *p* = 0.023). D-dimer level is a marker of activation of coagulation and fibrinolysis. However, in our study, the predictive score of D-dimers for thromboembolic events was not significant (ROC test, AUC = 0.79, *p* = 0.003, *n* = 116) compared to LOX-1-expressing neutrophil abundance in the blood (AUC = 0.89, *p* < 0.0001, *n* = 118). These results suggest that the high LOX-1 expression by ImNs might be a useful tool for predicting thromboembolic events among critically ill COVID-19 patients. The overexpression of LOX-1 may also be found in other cell types that might trigger the prothrombotic ERK1/2 pathway. The polymorphic *LOX-1* gene is also intensively associated with increased susceptibility to myocardial diseases. LOX-1 should thus be considered a potential target for therapeutic interventions.

The first step in viral entry of SARS-CoV-2 into host cells is binding of a viral envelope protein, known as the SPIKE (S) protein, to a cell host cell membrane receptor-enzyme protein, namely the angiotensin-converting enzyme 2 (ACE2) protein. ACE2 converts Angiotensin II (ANGII) the main effector of the renin-angiotensin system (RAS) to the vasodilator ANG1-7, which binds to MAS, a receptor that signals to reduce the activity of the ANGII receptor AT1R. Because ACE2 functions as both a SARS-CoV-2 receptor and a RAS regulator, the potential interference between of SARS-CoV-2 infection and RAS-controlled pathophysiology such as in heart, kidney and liver diseases was suspected. It has been suggested that the activation of the RAS could pre-dispose the patients with comorbidities to severe COVID-19 (45). Recently, patients with pre-existing cardiovascular diseases have a higher mortality rate and are five-times more likely to experience severe COVID-19 symptoms. LOX-1 is one of the major receptors of ox-LDL and thus an additional player in RAS (46). Dyslipidemia up-regulates and activates AT1R, and RAS activation in turn up-regulates and activates LOX-1, and then facilitates uptake of ox-LDL into endothelial cells. Activation of both receptors may predispose patients to severe COVID-19 infection. In this context, it is worth noting that plasma ox-LDL and LOX-1 messengers were increased in children with Kawasaki Disease (KD). Of note, recent clinical data suggest that SARS-CoV-2 may cause a pediatric multisystem inflammatory syndrome reminiscent of Kawasaki Disease (KD) in children (47).

In conclusion, we outline new measurable potential biomarkers of COVID-19 severity among immature circulating neutrophils: CD123, LOX-1, and PD-L1 surface markers. These markers are significantly correlated with disease severity, and even more so with thromboembolic events in the case of LOX-1.

## Data Availability Statement

The original contributions presented in the study are included in the article/**Supplementary Material**. Further inquiries can be directed to the corresponding authors.

## Ethics Statement

The studies involving human participants were reviewed and approved by local ethical committee of Sorbonne Université/Assistance Publique – Hôpitaux de Paris for standard hospitalized patients (N°2020-CER2020-21) and ICU patients (N° CER-2020-31). The patients/participants provided their written informed consent to participate in this study.

## Author Contributions

BC and CC designed the study and performed data analysis. LA, PR, NG, CP, OB, and AB performed experimental work and compiled and analyzed the data. PQ was responsible for clinical data mining and analysis. KD and DS performed cytokine dosage and analysis. MM, ZA, JM, C-EL, GG, and AG provided patient sample access. GG, AG, BC, and CC provided financial support. BC, LA, PQ, and CC wrote the manuscript. All authors contributed to the article and approved the submitted version.

## Funding

The study was supported by the Fondation de France, « Tous unis contre le virus » framework Alliance (Fondation de France, AP-HP, Institut Pasteur) in collaboration with the Agence nationale de la Recherche (ANR Flash COVID19 program ICOVID and ANR COV7 program Neutrosets), by the Programme Hospitalier de Recherche Clinique PHRC-20-0375 COVID-19, and by the SARS-CoV-2 Program of the Faculty of Medicine at Sorbonne University (I-COVID programs). The program was supported by AG2R-Région Ile de France. LA and PR are recipients of postdoctoral fellowships from the European Union’s Horizon 2020 research and innovation program funding, under grant agreement No. 681137.

## Conflict of Interest

The authors declare that the research was conducted in the absence of any commercial or financial relationships that could be construed as a potential conflict of interest.

## Publisher’s Note

All claims expressed in this article are solely those of the authors and do not necessarily represent those of their affiliated organizations, or those of the publisher, the editors and the reviewers. Any product that may be evaluated in this article, or claim that may be made by its manufacturer, is not guaranteed or endorsed by the publisher.
