# Supplementary material for: LOX-1-Expressing Immature Neutrophils Identify Critically-Ill COVID-19 Patients at Risk of Thrombotic Complications
Source: Front Immunol. 2021 Sep 20;12:752612. doi: 10.3389/fimmu.2021.752612 (PMC8488276; doi:10.3389/fimmu.2021.752612)
Supplement: Supplementary file 1 [file DataSheet_1.pdf]

LOX-1-expressing immature neutrophils identify critically-ill COVID-19 patients at risk of thrombotic complications

Behazine Combadière,[1,†,¥] Lucille Adam,[1,*] Noelline Guillou,[1,*] Paul Quentric,[1,2,*] Pierre Rosenbaum,[1] Karim Dorgham,[1] Olivia Bonduelle,[1] Christophe Parizot,[1,2] Delphine Sauce,[1] Julien Mayaux,[3] Charles-Edouard Luyt,[4,5] Alexandre Boissonnas,[1] Zahir Amoura,[6] Valérie Pourcher,[7] Makoto Miyara,[1,2] Guy Gorochov,[1, 2,†] Amélie Guihot,[1,2,†] and Christophe Combadière[1,†, ¥]

Supplementary figures

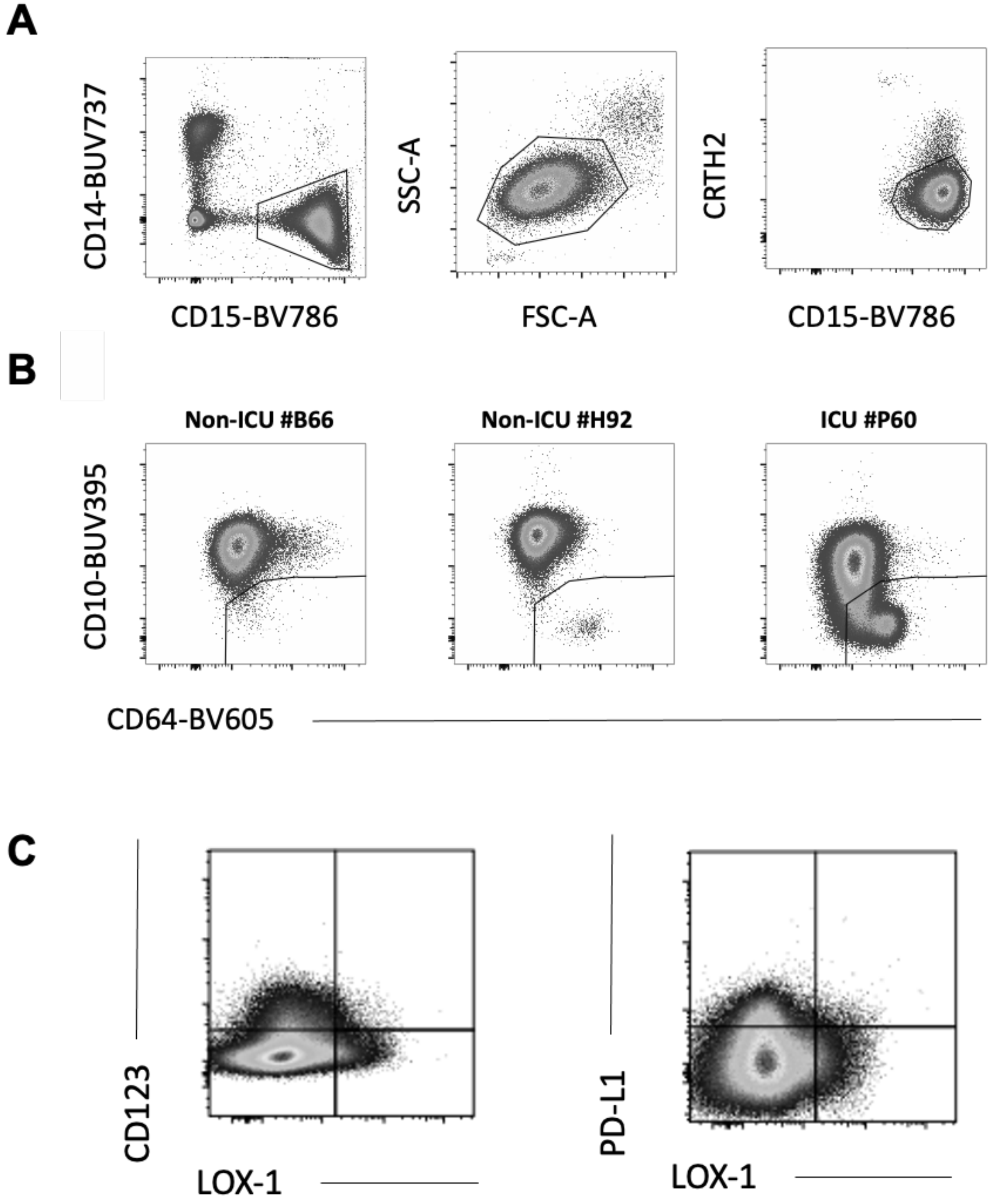

Supplementary Figure S1: Gating strategy used for the analysis of neutrophil populations by flow cytometry. (**A**) After debris and doublet exclusion, CD15+CD14− cells were selected. Eosinophils were excluded through side scatter (SSC) or forward scatter (FSC) parameters and the expression of CRTH2. The proportion of immature neutrophils was evaluated through the expression of CD10 and CD64. (**B**) One representative uninfected donor (#B66), COVID-19 non-ICU patient (#H92), and COVID-19 ICU patient (#60) are shown as an example. (**C**) Representative expression of LOX-1 and CD123 (left panel) or LOX-1 and PDL-1 (right panel) on CD10−CD64+ neutrophils (#ICU-G62).

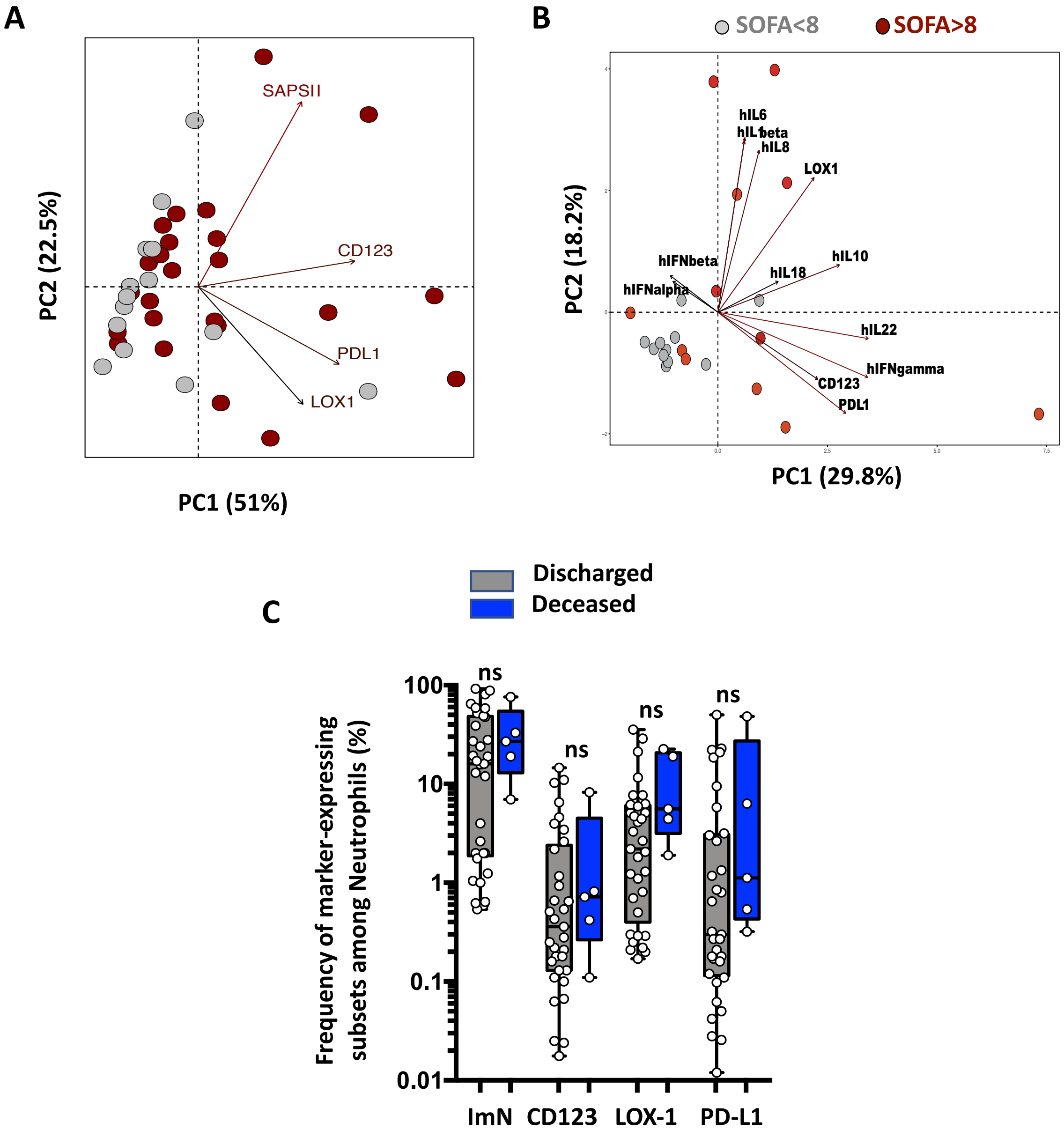

Supplementary Figure S2: Cytokine profiles and ImN subsets abundances associated with disease severity(**A**) Principal component analysis (PCA) using LOX-1+, PD-L1, CD123+, and CD10−CD64+ neutrophil abundance and Simplified Acute Physiology Score (SAPS) II variables on sample size: ICU = 24 (dark red circles) and non-ICU = 14 (gray circles). Percent contribution of each variable is indicated in color gradient black–red of the arrows. (**B**) PCA using serum cytokines and Sequential Organ Failure Assessment (SOFA) score variables on ICU patient sample size: high SOFA score (n = 11) and low SOFA score (n = 10) (SOFA <8 = gray circles; SOFA ≥8 = red circles). (**C**) Box plots (min to max distribution) of the abundance of CD10−CD64+ neutrophil subsets among total neutrophils of group samples of discharged (n = 33) and deceased (n = 5) patients.

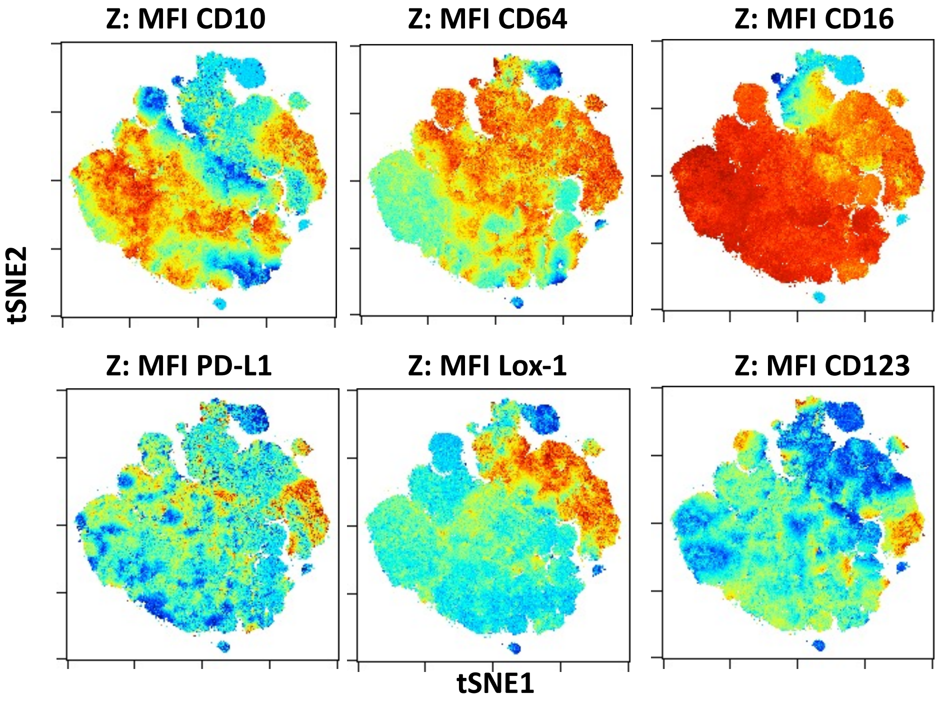

Supplementary Figure S3: Expression of neutrophil markers on a rainbow heat scale on the Opt-SNE map of the concatenated files of all clusters. The color gradient indicates high expression (red) to low expression (blue) of indicated Z markers.

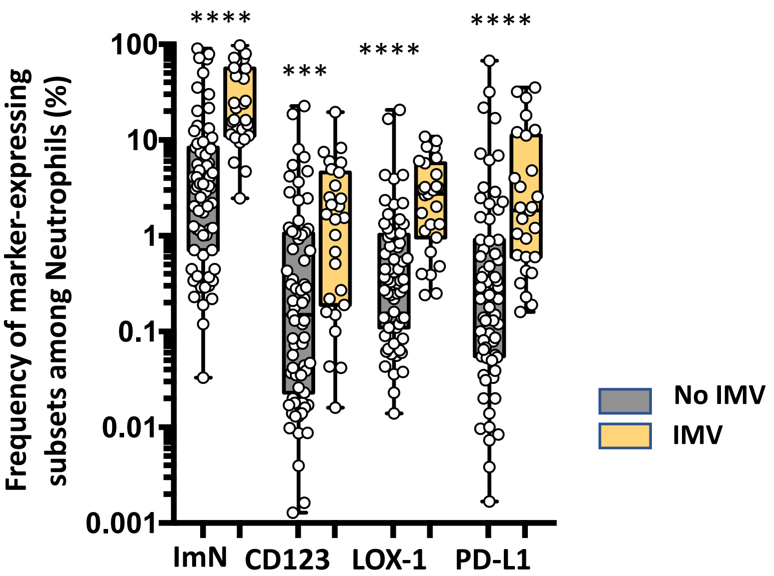

Supplementary Figure S4: Box plots (min to max distribution) of the abundance of CD10−CD64+ neutrophil subsets among patients requiring or not requiring invasive mechanical ventilation (no IMV: *n* = 60; IMV: *n* = 28). Nonparametric Mann–Whitney test was used to compare differences in cellular abundance of neutrophil subsets between groups, with significance defined by a *p*-value of *** for $p < 0.001$ and **** for $p < 0.0001$.

LOX-1-expressing immature neutrophils identify critically-ill COVID-19 patients at risk of thrombotic complications

Behazine Combadière,[1,†,¥] Lucille Adam,[1,*] Noelline Guillou,[1,*] Paul Quentric,[1,2,*] Pierre Rosenbaum,[1] Karim Dorgham,[1] Olivia Bonduelle,[1] Christophe Parizot,[1,2] Delphine Sauce,[1] Julien Mayaux,[3] Charles-Edouard Luyt,[4,5] Alexandre Boissonnas,[1] Zahir Amoura,[6] Valérie Pourcher,[7] Makoto Miyara,[1,2] Guy Gorochov,[1, 2,†] Amélie Guihot,[1,2,†] and Christophe Combadière[1,†, ¥]

Supplementary results

Demographics and baseline characteristics of ICU and non-ICU COVID-19 patients from the first wave

The first discovery study included 38 COVID-19 patients admitted to either ICU departments or non-ICU departments. Clinical and biological characteristics of the 38 patients are shown in Table 1. The median age of the patients was 57 years (range 25–79 years), and 65.8% were male. Analysis was performed on average 8 days after the onset of symptoms (median was 8 days for the ICU patients and 13 days for the non-ICU patients). The most common past medical comorbidities were hypertension (50%), type 2 diabetes (34.2%), and obesity (36.8%). The treatment regimen at baseline was mostly antihypertensive therapy (ACE inhibitors, 26.3%; angiotensin II receptor blockers, 15.8%). Severity at baseline was assessed by the SAPS II score for all patients (median 33; range 25–78) and an additional SOFA score for ICU patients (median 8.5; range 2–17). Twenty-eight patients were assessed with CT chest imaging, which showed ground-glass opacities and/or consolidation of >50% of the lung field in 50% of all patients, and up to 81.3% of the ICU patients. Laboratory findings showed a decreased median lymphocyte count at 0.94 × 10^9/L, an increased median neutrophil count at 7.87 × 10^9/L, an increased median lactate dehydrogenase at 475.5 U/L, and an increased median D-dimer level at 2450 ng/mL. During hospitalization, eight patients received hydroxychloroquine (42.1%), while all patients received antibiotics. Oxygen therapy was administered to 100% of the patients; 87.5% of ICU patients received invasive mechanical ventilation, while 54.2% received

extracorporeal membrane oxygenation. Acute respiratory distress syndrome occurred in 55.3% of all patients (87.5% of ICU patients); acute kidney injury, in 31.2% of all patients. Among the 38 patients, 2 were diagnosed with pulmonary embolism (5.3%) and 10 (all ICU) (26.3%) were diagnosed with venous thromboembolism. As of June 8, 2020, 76.3% of all patients had been discharged, 10.5% remained in hospital, and 13.2% had died, the latter all being ICU patients.

Demographics and baseline characteristics of ICU and non-ICU COVID-19 patients from the second wave

From mid-September 2020, our study included 118 COVID-19 patients admitted to either ICU departments or non-ICU departments. Clinical and biological characteristics of the 118 patients are shown in Table 2. Severity was defined according to WHO classification (https://www.who.int/publications/i/item/clinical-management-of-covid-19). Baseline clinical characteristics were similar to those of the first wave: median age was 61 years (range 21–78 years), with 63.6% being male, and there was a high prevalence of hypertension (52.5%), obesity (31.4%), and type 2 diabetes (30.5%). Analysis was performed on average 7 days after the onset of symptoms (7 days for the ICU patients; 5 days for the non-ICU patients). It is of note that these patients benefited from the last recommendations regarding standard of care, including corticosteroids and thromboprophylaxis, leading to an otherwise shorter duration in hospital, mostly among non-ICU patients.

LOX-1-expressing immature neutrophils identify critically-ill COVID-19 patients at risk of thrombotic complications

Behazine Combadière,[1,†,¥] Lucille Adam,[1,*] Noelline Guillou,[1,*] Paul Quentric,[1,2,*] Pierre Rosenbaum,[1] Karim Dorgham,[1] Olivia Bonduelle,[1] Christophe Parizot,[1,2] Delphine Sauce,[1] Julien Mayaux,[3] Charles-Edouard Luyt,[4,5] Alexandre Boissonnas,[1] Zahir Amoura,[6] Valérie Pourcher,[7] Makoto Miyara,[1,2] Guy Gorochov,[1, 2,†] Amélie Guihot,[1,2,†] and Christophe Combadière[1,†, ¥]

Supplementary Table S1

KEY RESOURCES TABLE

REAGENT or RESOURCE	SOURCE	IDENTIFIER
**Antibodies**		
CRTH2 FITC	Biolegend	#350107
CD123 PE	Biolegend	#306005
LOX1 BV421	Biolegend	#358609
CD64 BV605	Biolegend	#305033
PDL1 BV711	Biolegend	#329721
CD15 BV786	BD	#741013
CD14 BUV737	BD	#564444
CD10 BUV395	BD	#565975
Brillant violet buffer	BD	#563794
**Critical Commercial Assays**		
Human CorPlex™ Cytokine Panel 7-Plex array	Quanterix	# 85-0410
Simoa™ Human IL-3 Discovery Kit	Quanterix	#102462
Simoa™ IL-17A Advantage Kit	Quanterix	#101599
Simoa™ IL-18 Discovery Kit	Quanterix	#102700
Simoa™ GM-CSF Advantage Kit	Quanterix	#102329
Simoa™ Human IFN-a Advantage Kit	Quanterix	#100860
VeriKine-HS™ Human IFN Beta ELISA Kit	PBL Assay Science	#41415
**Software and Algorithms**		
RStudio version 1.3.959 Mac	Opensource	https://rstudio.com/
Cytobank	Cytobank	https://inserm.cytobank.org/
Prism version 8.00	Graphpad	https://www.graphpad.com/
SP-X Analysis Software	BD	https://www.quanterix.com/
Simoa HD-1 Software	Quanterix	https://www.flowjo.com/
